# Supplementary material for: XX sex chromosome complement promotes atherosclerosis in mice
Source: Nat Commun. 2019 Jun 14;10:2631. doi: 10.1038/s41467-019-10462-z (PMC6643208; doi:10.1038/s41467-019-10462-z)
Supplement: Supplementary file 1 — Supplementary Information [file 41467_2019_10462_MOESM1_ESM.docx]

Supplementary Information

**XX sex chromosome complement promotes atherosclerosis in mice**

Yasir AlSiraj^1^, Xuqi Chen^2^, Sean E. Thatcher^1^, Ryan Temel^1,3,4^, Lei Cai^1,3^, Eric Blalock^1^, Wendy Katz^1^, Heba M. Ali^1^, Michael C. Petriello^5^, Pan Deng^5^, Andrew Morris^5^, Xuping Wang^6,7,8^, Aldons J. Lusis^6,7,8^, Katherine Thompson^9^, Arthur P. Arnold^2^, Karen Reue^8^, Patrick Tso^10^, Lisa A. Cassis^1*^

^1^Department of Pharmacology and Nutritional Sciences, University of Kentucky, Lexington KY; ^2^Department of Physiology, University of Kentucky, ^3^Integrative Biology and Physiology, University of California, Los Angeles CA; ^4^Saha Cardiovascular Research Center, University of Kentucky, Lexington, KY; ^5^Division of Cardiovascular Medicine, Department of Internal Medicine, University of Kentucky, and Lexington Veterans Affairs Medical Center, Lexington KY; ^6^Medicine, University of California, Los Angeles; ^7^Microbiology, Immunology and Molecular Genetics, University of California, Los Angeles; ^8^Human Genetics, University of California, Los Angeles; ^9^Department of Statistics, University of Kentucky; ^10^Department of Pathology, University of Cincinnati, Cincinnati OH

**A**

**B**

C

**Supplemental Figure 1**. Triglyceride (A) or cholesterol (B) concentrations in livers from GDX XX and XY male and female *Ldlr^-/-^* mice fed a Western diet for 4 months. C, Tissue sections from livers of mice from each group. Symbols represent individual mice, with mean ± SEM illustrated by horizontal lines. Scale bar = 100 μm.

**Supplemental Figure 2.** Hepatic VLDL secretion rates (plasma TG over time, A), slope of A (beginning from 1 hour, B), accumulation of newly synthesized and secreted apolipoprotein B100 (ApoB100) (3 hour time point from (A)) (C), quantification of apolipoprotein B48 (ApoB48) levels in C (D), quantification of ApoB100 levels in C (E). Symbols represent individual mice. Data are mean ± SEM (horizontal lines) from n = 5 mice/group. #, P<0.05 compared to female within genotype. @, P<0.05 XX versus XY (overall sex chromosome effect).

**
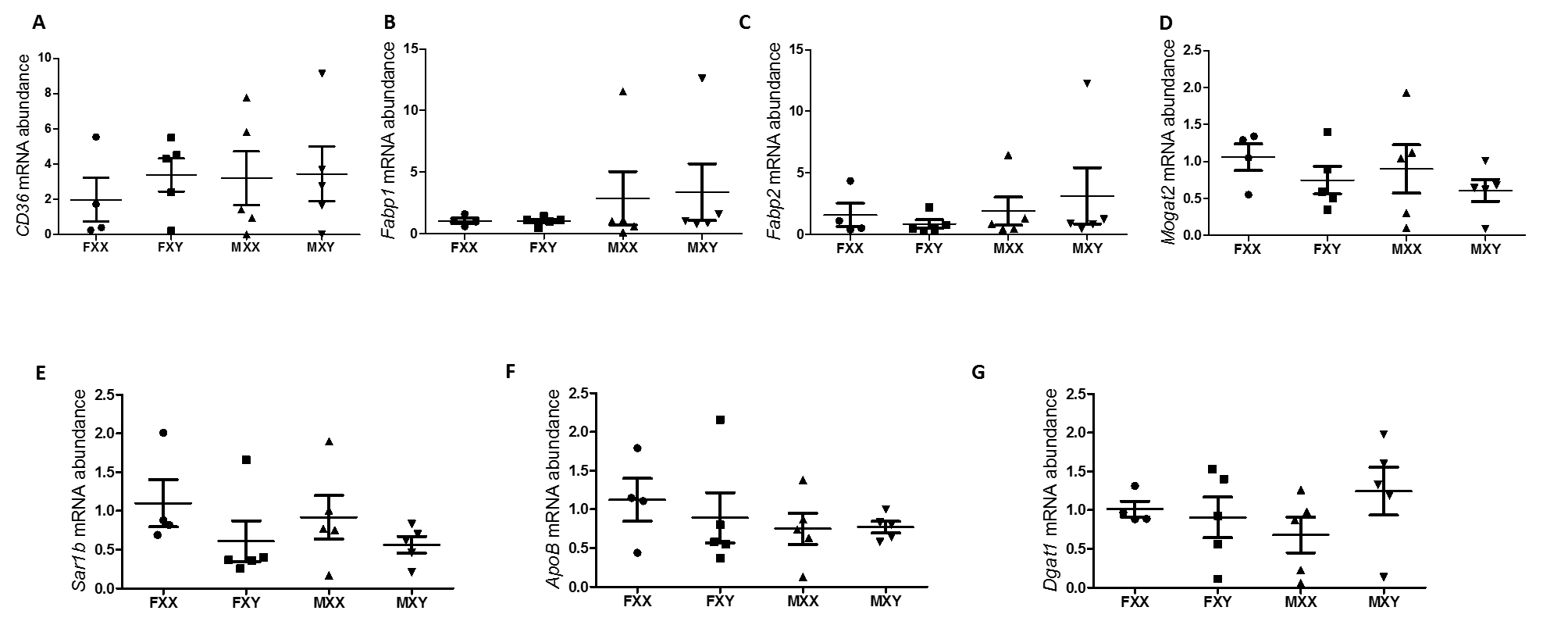
**

**Supplemental Figure 3**. mRNA abundance of *Cd36* (A), *Fabp1* (B), *Fabp2* (C), *Mogat2* (D), *Sar1b* (E), *Apob* (F), and *Dgat1* in small intestines from GDX XX compared to XY female and male *Ldlr^-/-^* mice. Symbols represent individual mice. Data are mean ± SEM (horizontal lines) from n = 4-5 mice/group.


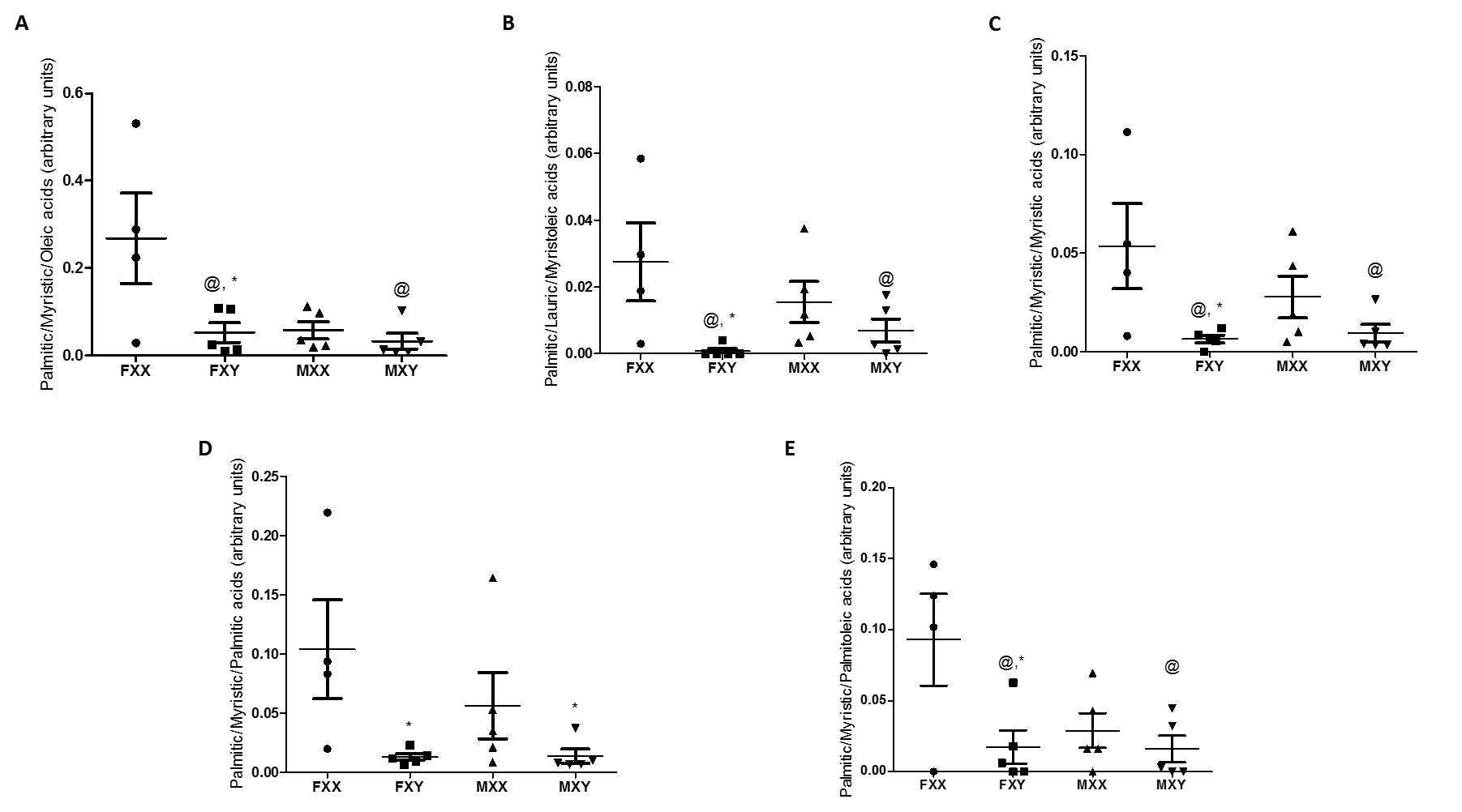


**Supplemental Figure 4**. Fatty acid mixtures in small intestines from four core genotype (FCG) XX compared to XY female and male GDX *Ldlr^-/-^* mice. Symbols are individual mice. Data are mean ± SEM (horizontal lines) from n = 4-5 mice/group. *, P <0.05 compared to XX within gonadal sex. @, P<0.05 XX *versus* XY (overall sex chromosome effect).

**B**

**C**

**A**

**D**

**Supplemental Figure 5**. Microbial alpha diversity as determined by Chao1 (A), PD whole tree (B) observed OTU indices (C) and Shannon Diversity Index (D) from male and female XX and XY four core genotype (FCG) *Ldlr^-/-^* mice fed the Western diet for 4 weeks. Symbols are individual mice. Data are mean ± SEM (horizontal lines) from n = 4-6 mice/group. @, P<0.05 females versus males (overall sex effect).

| **Gene** | **Forward primer** | **Reverse primer** |
| --- | --- | --- |
| *Apob* | Cagtattctgccactgcaacc | aggacttcactagataaggtcc |
| *Cd36* | Gagactgggaccattggtga | tatatgtaggctcatccactac |
| *Fabp1* | Gtcaaggcagtcgtcaagct | tcttgtagacaatgtcgccca |
| *Fabp2* | Tagaccggaacgagaactatg | aatggtccaggccccagtga |
| *Mogat2* | Tggtggtacctggactgggaca | Gtggaagcccgcgatgtagttc |
| *Sar1b* | CAGCACGTCCCAACGCTACATC | ACGTGCCCACCCAGATCAAAA |
| *DGAT2* | CCGCAAAGGCTTTGTGAAG | GGAATAAGTGGGAACCAGATCA |
| *MTTP* | TGGAGCCCTGGTCAGGAA | CCACTGCCTTGAGCTTGCA |
| *Beta actin* | GCTCTGGCTCCTAGCACCAT | GCCACCGATCCACACAGAGT |
| *Beta2 microglobulin* | GGCCTGTATGCTATCCAGAA | GAAAGACCAGTCCTTGCTGA |
| *Gapdh* | GCCAAAAGGGTCATCATCTC | GGCCATCCACAGTCTTCT |

**Supplemental Table 1**. Primers for RT-PCR.
