# Supplementary material for: XX sex chromosome complement promotes atherosclerosis in mice
Source: Nat Commun. 2019 Jun 14;10:2631. doi: 10.1038/s41467-019-10462-z (PMC6643208; doi:10.1038/s41467-019-10462-z)
Supplement: Supplementary file 3 — Description of Additional Supplementary Files [file 41467_2019_10462_MOESM3_ESM.docx]

**Description of Supplementary Files**

**File Name:** Supplementary Data 1

**Description:** Significant genes are listed in alphabetical order by gene symbol. Columns: Probe set ID- Affymetrix-based transcript-level probe-set identification numbers; Chromosome- chromosome location annotation; Symbol- official gene symbol annotation; #p- number of samples in which signal intensity was > 4.2 ; Mean + SEM (standard error of the mean) [for FX- gonadal female, XX genotype; FY- gonadal female, XY genotype; MX- gonadal male, XX genotype; MY- gonadal male, XY genotype]; ANOVA- analysis of variance p-values- probability values; q-values- multiple testing-corrected p-values [for Sex- main effect of gonadal sex; Chrom- main effect of chromosomal complement; Intrxn- Interaction term]; L2FCs- Log 2 fold change comparisons (Fgonadal female; M- gonadal male; X- XX chromosomal complement; Y- XY chromosomal complement). Note: for L2FCs, a negative value indicates that the column label denominator value is larger, and a positive L2FC indicates that the column label numerator value is larger.
