# Supplementary material for: XX sex chromosome complement promotes atherosclerosis in mice
Source: Nat Commun. 2019 Jun 14;10:2631. doi: 10.1038/s41467-019-10462-z (PMC6643208; doi:10.1038/s41467-019-10462-z)
Supplement: Supplementary file 5 — Reporting Summary [file 41467_2019_10462_MOESM5_ESM.pdf]

nature research

Corresponding author(s): Lisa A. Cassis

Last updated by author(s): May 8, 2019

Reporting Summary

Nature Research wishes to improve the reproducibility of the work that we publish. This form provides structure for consistency and transparency in reporting. For further information on Nature Research policies, see Authors & Referees and the Editorial Policy Checklist.

Statistics

For all statistical analyses, confirm that the following items are present in the figure legend, table legend, main text, or Methods section.

n/a	Confirmed	
☐	☒	The exact sample size (*n*) for each experimental group/condition, given as a discrete number and unit of measurement
☐	☒	A statement on whether measurements were taken from distinct samples or whether the same sample was measured repeatedly
☐	☒	The statistical test(s) used AND whether they are one- or two-sided<br>*Only common tests should be described solely by name; describe more complex techniques in the Methods section.*
☐	☒	A description of all covariates tested
☐	☒	A description of any assumptions or corrections, such as tests of normality and adjustment for multiple comparisons
☐	☒	A full description of the statistical parameters including central tendency (e.g. means) or other basic estimates (e.g. regression coefficient) AND variation (e.g. standard deviation) or associated estimates of uncertainty (e.g. confidence intervals)
☐	☒	For null hypothesis testing, the test statistic (e.g. *F*, *t*, *r*) with confidence intervals, effect sizes, degrees of freedom and *P* value noted<br>*Give P values as exact values whenever suitable.*
☒	☐	For Bayesian analysis, information on the choice of priors and Markov chain Monte Carlo settings
☒	☐	For hierarchical and complex designs, identification of the appropriate level for tests and full reporting of outcomes
☒	☐	Estimates of effect sizes (e.g. Cohen's *d*, Pearson's *r*), indicating how they were calculated

Our web collection on statistics for biologists contains articles on many of the points above.

Software and code

Policy information about availability of computer code

Data collection	N/A
Data analysis	Graphpad Prism, SigmaStat, R [Version 3.4.0]

For manuscripts utilizing custom algorithms or software that are central to the research but not yet described in published literature, software must be made available to editors/reviewers. We strongly encourage code deposition in a community repository (e.g. GitHub). See the Nature Research guidelines for submitting code & software for further information.

Data

Policy information about availability of data

All manuscripts must include a data availability statement. This statement should provide the following information, where applicable:

- Accession codes, unique identifiers, or web links for publicly available datasets
- A list of figures that have associated raw data
- A description of any restrictions on data availability

All data are available from the corresponding author upon reasonable request. Raw microarray data (Figure 4) are available through the Gene Expression Omnibus under the accession code GSE119497 [https://www.ncbi.nlm.nih.gov/geo/query/acc.cgi?acc=GSE119497]. The source data underlying Figures 1a, 2a-d, 6d, h and 7c and Supplementary Figures 1a and 5d are provided as a Source Data file.

Field-specific reporting

Please select the one below that is the best fit for your research. If you are not sure, read the appropriate sections before making your selection.

☒ Life sciences ☐ Behavioural & social sciences ☐ Ecological, evolutionary & environmental sciences

For a reference copy of the document with all sections, see nature.com/documents/nr-reporting-summary-flat.pdf

Life sciences study design

All studies must disclose on these points even when the disclosure is negative.

Sample size	We performed a power analysis on atherosclerosis as the primary endpoint in previous studies, and have used this to guide the sample size for studies with this as the primary endpoint.
Data exclusions	There are no data exclusions.
Replication	For atherosclerosis, lesions were quantified by 2 observers blinded to the study design. We use housekeeping genes, which are tested for between group differences, to normalize gene expression data from RT-PCR. We use internal standards for mass spectroscopy analysis.
Randomization	Within an experimental group (e.g., genotype), allocation to groups were random.
Blinding	Mice were assigned numbers without group identification. Measurements were performed for each sample number, and for some measurements, two observers obtained results.

Reporting for specific materials, systems and methods

We require information from authors about some types of materials, experimental systems and methods used in many studies. Here, indicate whether each material, system or method listed is relevant to your study. If you are not sure if a list item applies to your research, read the appropriate section before selecting a response.

Materials & experimental systems

n/a	Involved in the study	
☐	☒	Antibodies
☒	☐	Eukaryotic cell lines
☒	☐	Palaeontology
☐	☒	Animals and other organisms
☒	☐	Human research participants
☒	☐	Clinical data

Methods

n/a	Involved in the study	
☒	☐	ChIP-seq
☒	☐	Flow cytometry
☒	☐	MRI-based neuroimaging

Antibodies

Antibodies used	Goat anti-human apoB antiserum; Academy Bio-Medical Co, Houston, TX, USA Cat #205-G2
Validation	http://www.ncbi.nim.nih/gov/pmc/articles/PMC3880293/#pone.0084418-Chung1<br>https://www.ncbi.nim.nih.gov/pmc/articles/PMC2852959/

Animals and other organisms

Policy information about studies involving animals; ARRIVE guidelines recommended for reporting animal research

Laboratory animals	Male and female mice (low density lipoprotein deficient, apolipoprotein E deficient, C57BL/6J), 3-4 months of age
Wild animals	N/A
Field-collected samples	N/A
Ethics oversight	All experiments were approved by the animal care and use committee at the University of Kentucky and the University of California, Los Angeles and conformed to the Guide for the Care and Use of Laboratory Animals published by the NIH.

Note that full information on the approval of the study protocol must also be provided in the manuscript.
